# Recent Advances in Allergy Research Using Humanized Mice

**DOI:** 10.3390/ijms20112740

**Published:** 2019-06-04

**Authors:** Ryoji Ito, Shuichiro Maruoka, Yasuhiro Gon, Ikumi Katano, Takeshi Takahashi, Mamoru Ito, Kenji Izuhara, Satoshi Nunomura

**Affiliations:** 1 Central Institute for Experimental Animals (CIEA), Kawasaki 210-0821, Japan; katano-i@ciea.or.jp (I.K.); takeshi-takahashi@ciea.or.jp (T.T.); mito@ciea.or.jp (M.I.); 2 Division of Respiratory Medicine, Nihon University School of Medicine, Tokyo 173-8610, Japan; maruoka.shuichiro@nihon-u.ac.jp (S.M.); gon.yasuhiro@nihon-u.ac.jp (Y.G.); 3 Division of Medical Biochemistry, Department of Biomolecular Sciences, Saga Medical School, Saga 849-0937, Japan; nunomura@cc.saga-u.ac.jp

**Keywords:** airway inflammation, allergy, disease model, eosinophil, humanized mouse

## Abstract

The prevalence rates of allergic diseases are increasing worldwide, particularly in industrial countries. To date, many mouse models have been generated for allergy research; studies conducted using these models have suggested the importance of cross-talk between immune cells and tissue-resident non-immune cells in the onset of allergic diseases. However, there are several differences between the immune systems of rodents and humans, and human studies are limited. Thus, mice reconstituted with human immune cells are a novel tool for the preclinical evaluation of the efficacy and safety of developing drugs. Genetic technologies for generating humanized mice have improved markedly in recent years. In this review, we will discuss recent progress in allergy research using humanized mice and introduce our recent humanized mouse model of airway inflammation in human immune cells.

## 1. Introduction

The development of model animals has greatly contributed to progress in basic research on physiological mechanisms in humans. Rodent models are the gold standard in most fields of biomedical research due to their small size, large litters, availability of many inbred strains, and their ease of maintenance, handling, and reproductive engineering. Because exogenous DNA is easily integrated into the murine genome, genetic modification techniques including knockout, knockin, and transgenic techniques were initially established using fertilized mouse eggs or embryonic stem cells [1,2,3,4]. These techniques enabled the clarification of the physiological functions of numerous genes and generated many important models that mimic the pathogenesis of human diseases. However, murine models cannot fully recapitulate the physiological and pathological mechanisms underlying most human diseases because the expression level or functional status of the molecules frequently differ between human and rodent immune cells.

Human and mouse hematopoietic stem cells (HSC) can differentiate into a variety of granulocyte subsets, such as eosinophils, basophils, and mast cells [5,6]. However, morphological and functional differences in these myeloid-lineage cells had been reported between humans and mice. Morphologically, both mature human and mouse eosinophils have polymorphic nuclei. However, in human eosinophils, the nuclei have multiple lobes. Moreover, human eosinophils stain a more vibrant magenta with eosin than mouse eosinophils due to the higher cationic change of the human granule proteins [6]. Human basophils exhibit a more granulated phenotype compared to mouse basophils [7]. The high-affinity IgE receptor for IgE (FcεRI) is responsible for IgE-mediated allergic reactions. Human mast cells, basophils, and activated eosinophils express FcεRI as αβγ2 tetramers or αγ2 trimers. By contrast, mouse eosinophils do not express FcεRI on the cell surface [8]. This implies that FcεRI crosslinking with IgE and allergens induces partially restricted allergic responses in the mouse asthma model. Moreover, human IgE does not induce an immune response via mouse FcεRI in mice, which implies that mouse strains are not suitable for testing human IgE-mediated biological responses such as passive cutaneous anaphylaxis (PCA). Passive systemic anaphylaxis (PSA) and active systemic anaphylaxis (ASA) are also inducible in mice in an IgG1-dependent manner. Murine IgG1 antibodies and macrophages play important roles in the development of ASA elicited by epicutaneous peanut sensitization and antigen challenges [9]. It is unclear how human IgG1 contributes to anaphylaxis; however, there are certain differences in biological activity between human and mouse IgG1 antibodies. For example, mouse IgG1 functionally resembles human IgG4 rather than human IgG1 [10]. These two Igs fail to activate in response to complement C1q, which is converted to anaphylatoxins. Regarding IgA antibodies, most IgA-mediated eosinophil activation requires interactions with IgA receptors. CD89 has been cloned from activated human monocytes and identified as the Fc receptor for IgA [11]. Decot et al. demonstrated that human eosinophils express functional CD89, while mouse eosinophils do not [12]. Therefore, mouse eosinophils are thought to be hyporesponsive to stimulation via FcεRI and CD89. The exocytosis of pre-stored mediators from granulocytes in response to external stimuli, including the cross-linking of Fc receptors, is essential for the development of allergic responses. Among these three granulocytes, the eosinophil degranulation response differs markedly between humans and mice. Studies of both human patients and mouse models have demonstrated that piecemeal degranulation is a common mechanism of the exocytosis of eosinophilic granule contents and occurs in vivo in the airways of asthmatic patients. By contrast, researchers have not detected eosinophil degranulation in mouse airways. Stelts et al. reported that mouse airway eosinophils are exceedingly resistant to degranulation, even when repeatedly exposed to huge doses of allergen [13].

Nonhuman primates are closely related to humans and show high sequence homology with the human genome. Several human immune diseases might be accurately recapitulated by using nonhuman primate models, such as infectious, autoimmune, and allergic disease models [14,15,16,17]. Recently, our group established a severe combined immunodeficient (SCID) common marmoset in which genome editing technologies, such as zinc finger nuclease (ZFN) and transcription activators like effector nuclease (TALEN), were used to target the IL-2 receptor gamma chain [18]. However, it is difficult to use it as an experimental animal model for general purposes, because the use of nonhuman primates has several limitations, including ethical problems, high cost, low fertility, and difficulty of handling.

The discovery of immunodeficient mice, such as nude [19] and SCID [20] mice, in which human cancers, hematopoietic cells, skin, and other organs can be engrafted, was a major breakthrough in xenotransplant studies [21,22,23]. Such xenotransplant model mice are called ‘humanized mice’ and have been used to model various human diseases. This review focuses on the recent progress in humanized mouse models, including the generation of several strains of second-generation humanized mice and a model for human allergic diseases that was recapitulated using second-generation humanized mice

## 2. Development of First-Generation Humanized Mice

Humanized mice, produced by the reconstitution of human hematopoietic cells in immunodeficient mice, have facilitated the study of human hematology and immunology in vivo and allowed us to recapitulate human cell-mediated immune diseases. The past three decades have seen the development of humanized severe combined immunodeficient (SCID-hu) mice and human peripheral blood mononuclear cell (hu-PBMC) SCID mice [24,25]. *Prkdc*-mutated CB17-scid mice transplanted with fetal-derived human immune organs or PBMCs are innovative animal models for the reconstruction of human immune systems [20]. Non-obese diabetes (NOD) scid mice have also commonly been humanized for immune system research [26,27], because the NOD background exhibits two base-pair deletions in a complement C5 gene [28,29], combined with a specific signal regulatory protein α (Sirpα) polymorphism that can recognize the human CD47 ligand to induce macrophage tolerance against human transplants [30,31]. However, the potential capacity for xeno-engraftment in both CB17-scid and NOD-scid mice remains inefficient due to remaining murine innate immunity. An interleukin (IL)-2 receptor common gamma (IL-2rγ) subunit, which is the receptor for six cytokines, IL-2, IL-4, IL-7, IL-9, IL-15, and IL-21, plays an important role in both innate and acquired immunity [32,33], and its deficiency causes X-linked SCID in humans [34,35]. A significant breakthrough in the research field of humanized mice was the generation of NOD-scid mice harboring an Il2rγ gene knockout, which are referred to as NOD-scid IL2rγ^null^ (NOG or NSG) mice [36,37,38]. The NOG or NSG strain has severe multiple immunodeficiencies, including a lack of T, B, and natural killer (NK) cells and functional defects in macrophages and dendritic cells [39], and it therefore has high engraftment rates of human cancer cells [40,41,42,43,44], PBMCs [45,46], and hematopoietic stem cells (HSCs) [36,37,38]. Importantly, both NOG and NSG mouse strains exhibited dramatic improvements in the engraftment rate and hematopoiesis following CD34^+^ HSC transplantation, marked by the simultaneous appearance of numerous and mature human T and B cells in peripheral organs. To date, studies using humanized mice as a human disease model have focused on tumors or infectious diseases [47,48,49,50,51,52,53], because these types of diseases are more dependent on human lymphocytes, which are well-differentiated in conventional NOG or NSG mice. However, several problems—e.g., the poor differentiation of human myeloid and NK cells and acquired immunity dysfunction—limit the reconstitution of human immune systems in conventional humanized NOG or NSG mice. These problems could restrict the application of humanized mouse models to other immunological diseases. In the next section, we introduce improvements made to NOG or NSG mice to overcome these issues.

## 3. Development of Second-Generation Humanized Mice

We and other groups have introduced human cytokine genes into NOG, NSG, or BALB/cA background RAG2^null^ and IL-2rγ^null^ (BRG) mice to develop myeloid lineage cells [54,55,56,57,58,59], NK cells [60,61,62], and functional T cells [63,64]; we and others have partially succeeded in recapitulating human immunological diseases [65,66,67,68,69,70]. These mice are widely recognized as second-generation humanized mice. Previously, four strains of second-generation humanized mice were developed (Table 1), including human IL-3, granulocyte macrophage colony stimulating factor (GM-CSF), and stem cell factor (SCF) gene-introduced NSG mice (NSG SGM3), which are characterized by extended myelopoiesis [55,71]. These NSG SGM3 mice are used for studies of human disease such as patient-derived acute myeloid leukemia or myelodysplastic syndrome transplants [71,72], Ebola viral infection [73], and allergies [74]. Because these mice show a marked differentiation of human mast cells, passive cutaneous anaphylaxis (PCA) and passive systemic anaphylaxis (PSA) can be induced by administering human IgE antibodies and the antigen in vivo [74]. This strain also shows enhanced human CD4^+^FoxP3^+^ regulatory T (Treg) cell differentiation, and these Treg cells are functional and suppress the proliferation of T cells ex vivo [55]. MISTRG mice were established by introducing human macrophage colony-stimulating factor (M-CSF), IL-3, Sirpα, thrombopoietin, and GM-CSF genes into BRG mice [56]. This strain shows the robust differentiation of innate immune cells, including myeloid and NK cells, and has been used for engrafted patient-derived acute myeloid leukemia or myelodysplastic syndrome [75,76] and Listeria monocytegenes and influenza A virus infection [56]. Human myelopoiesis was also enhanced in MISTRG mice with HSC derived from G-CSF mobilized human PBMC, and those HSCs were effectively engrafted in them compared to conventional NSG mice [77]. NOG human IL-3 and GM-CSF transgenic (IL-3/GM-CSF Tg; also known as NOG EXL) mice [57] were developed by our research group; these mice exhibit multiple myelopoiesis, similar to that shown in NSG SGM3 and MISTRG mice. Due to increased numbers of CD4^+^ T and myeloid cells compared with non-transgenic NOG mice, humanized NOG IL-3/GM-CSF Tg mice have been shown to support human immunodeficiency virus (HIV) replication and recapitulate the hematopoietic conditions in patients [78]. Notably, human mast cell-mediated PCA and eosinophilic airway inflammation are inducible in NOG IL-3/GM-CSF Tg and NOG IL-3/GM-CSF Tg combined with human IL-5 Tg (NOG IL-3/GM-CSF/IL-5 Tg) mice [57,65]. Human myeloid lineage cells are also significantly differentiated in membrane-bound human SCF Tg mice [54,59]. The strain shows significant human mast cell differentiation and develops a food allergy induced by peanut butter [79]. Thus, second-generation humanized mice have facilitated the analysis of greater numbers of human immune cells and immune disease models than was previously possible. In the following sections, we focus on human allergic disease models using conventional or second-generation humanized mouse models and discuss their achievements, limitations, and potential applications in future studies.

## 4. Humanized Mouse Model for Allergy Research

### 4.1. PBMC-Transferred Allergy Models

**Figure 1:**
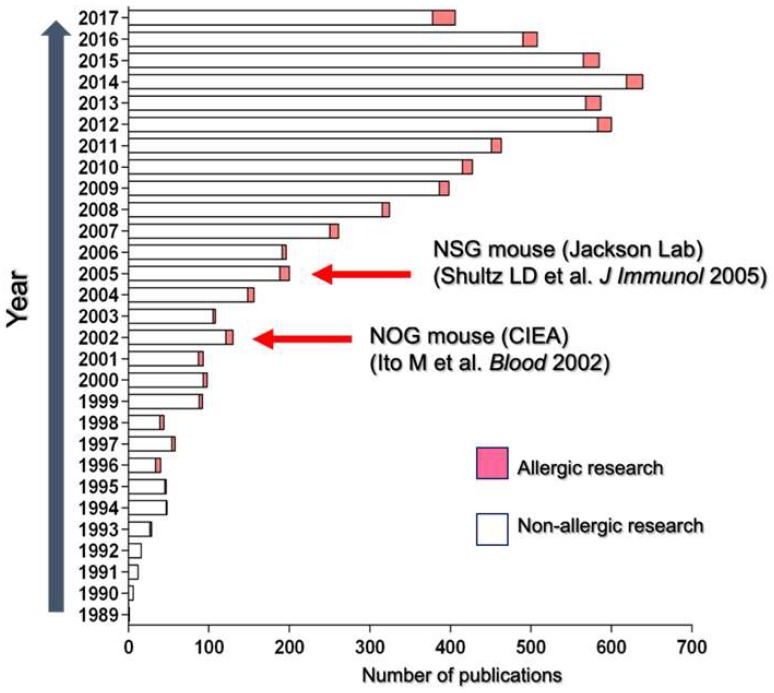
Publication history of humanized mouse research. The number of publications related to allergy (pink) and non-allergy (white) research using humanized mice between 1989 and 2017 are presented. Red arrows indicate the establishment of NOD-scid IL2rγ^null^ (NOG or NSG) mice.

An increasing number of publications in biomedical research fields have relied on humanized mice since the establishment of NOG or NSG mice (Figure 1). Though allergy research represents a small proportion of humanized mouse studies, the publication rate of human cell-mediated allergic reaction studies is gradually increasing. One reason for this increase is that the sequence homology among receptors for chemical mediators such as histamines, platelet-activating factors, and cysteinyl leukotriene 1 (CysLT1) is highly conserved between humans and rodents. Therefore, these human cell-produced chemical mediators can induce allergic reactions and recapitulate various allergic disorders [80,81]. Human PBMC-engrafted models have facilitated the recapitulation of human IgE-mediated allergic diseases [82,83,84,85,86,87,88]. Weigmann et al. [84] used NSG mice transplanted with PBMCs derived from allergy patients to demonstrate that colonic inflammation is induced by the rectal or oral challenge of allergens. Engrafted patient-derived B cells produced an allergen-specific IgE antibody in mice, and an allergic inflammation was induced by the administration of the specific allergens. This gut inflammation was suppressed by omalizumab, an anti-human IgE monoclonal antibody, suggesting the involvement of IgE- and FcεR-expressing cells in disease onset. Glycoprotein A repetitions predominant (GARP)-expressing Treg cells or recombinant GARP significantly inhibited this gut inflammation induced by allergen administration [89]. These models have used human PBMC-transferred humanized mice to gain new insight into therapeutic strategies for allergic gut inflammation.

Atopic dermatitis (AD) is a common chronic inflammatory skin disease mediated by T helper type 2 cells, an allergen-specific IgE, and type 2 cytokines (e.g., IL-4, IL-13, and IL-31). These mediators play a crucial role in developing skin inflammation and intolerable itching in pruritic eczema [90,91,92,93]. Hapten-induced dermatitis mouse models are commonly used in studies of AD; in these models, chronic inflammatory responses such as allergic contact dermatitis have been induced by T helper type 2 cells similar to those in human AD patients [94,95,96]. To recapitulate human AD pathophysiology, Nolte et al. [85] established oxazolone-induced skin inflammation in humanized mouse models. The authors transferred human PBMCs from AD patients into NSG mice and sensitized and challenged the skin with oxazolone. A repeated challenge with oxazolone induced epithelial hyperplasia, IgE secretion, and human T cell inflammation in the skin, similar to the characteristic features of AD patients.

Food allergies are common conditions induced by exposure to food-derived allergens such as egg, milk, wheat, and peanuts. Systemic anaphylaxis, generally mediated by degranulation in IgE–allergen complex-crosslinked mast cells, causes rapid death following the consumption of food containing allergens; such allergic reactions are a serious problem in food allergy patients [97,98,99,100]. Though murine food allergy models with anaphylaxis are commonly used worldwide and have been used to analyze the pathogenic mechanisms [101,102,103], there is only one recent model of food allergy using PBMC-transferred humanized mice. Pagovich et al. [86] developed a systemic anaphylaxis model induced by repetitive gastric gavage of a peanut extract into NSG mice transferred with human PBMCs from peanut allergy patients. Interestingly, anaphylactic pathology was successfully inhibited by gene therapy via the administration of an adeno-associated virus gene-transfer vector encoding anti-human IgE-neutralizing antibodies. Thus, a new approach to treat food allergies or any other IgE-mediated allergic disorders has been proposed.

### 4.2. Limitations of PBMC-Transferred Allergy Models

There are several limitations of using human PBMC-transferred models for allergy research. First, since human FcεR-expressing mast cells and basophils are rarely included among PBMCs, it is very difficult to induce histamine degranulation or leukotriene synthesis despite the production of allergen-specific human IgE from human B cells. Second, we previously demonstrated that CD4^+^ Th17 cells induced skin inflammation in NOG mice following the transfer of isolated human CD4^+^ T cells from PBMCs [104]. In this model, human Th17 cells were recruited to mouse skin by murine chemokines and activated to elicit inflammation by major histocompatibility complex (MHC)-mediated xenogenic mechanisms. Therefore, it may be difficult to clarify whether or not skin inflammation is induced by allergic responses, even when AD patient-derived PBMCs are used. Third, PBMC transplantation is an inadequate protocol for long-term evaluation, including that of therapies requiring multiple administrations of drugs due to severe xenogenic graft-versus-host disease [45,46]. Therefore, as described below, alternative models are necessary to develop practical allergy models for preclinical studies.

### 4.3. HSC-Transferred Allergy Model

Human mast cell- or basophil-reconstituted humanized mice are considered useful and reliable allergy models because these cells express abundant and various inflammatory mediators, such as histamine and leukotriene, and can elicit allergic reactions in response to external stimuli. Novel protocols to reconstitute human immune cells including mast cells and basophils have been established using second-generation humanized mice. NOG IL-3/GM-CSF Tg mice have allowed the development of human FcεRI-expressing mast cells and basophils after human HSC transplantation [57]. Using this model, we can induce human mast cell-mediated PCA reactions upon the stimulation of hapten-specific human IgE plus its hapten or serum containing Japanese cedar pollen-specific IgE antibodies from pollinosis patients. In this context, murine complement component 3 (C3) may contribute to human mast cell degranulation in humanized mice because the inhibition of murine C3, but not C5, suppresses PCA reactions. However, further study is required to elucidate the roles of murine C3 in PCA reactions via human mast cells.

NSG hSCF Tg mice also exhibit the predominant differentiation of human mast cells following HSC transplantation [54,59]. Burton et al. [79] developed peanut butter-induced food allergy models using HSC-humanized NSG hSCF Tg mice; they sensitized NSG hSCF Tg mice by the repeated oral administration of small amounts of peanut butter for eight weeks, and they then followed that by a challenge with a large amount of peanut butter. The mice showed characteristics of human mast cell-induced systemic anaphylaxis, such as decreased body temperature and the secretion of mast cell tryptase. Surprisingly, human T cells restricted by human MHC appeared, such that the mice produced peanut-specific human IgE and IgG in serum. These findings imply that in NSG hSCF Tg mice, active allergic reactions are the result of initial cognate interactions between human T and B cells. Omalizumab, an anti-human IgE antibody, was shown to inhibit these symptoms. Thus, second-generation humanized mice are useful for recapitulating mast cell-induced anaphylaxis and may have potential applications for the development of novel humanized antibodies against human IgE.

### 4.4. Bone Marrow, Liver, Thymus (BLT) Allergy Model

Human fetal bone marrow, liver, thymus (BLT)-transplanted immunodeficient mice (BLT mice) are humanized mice that reconstitute all lineages of human immune cells [105,106,107,108]. These mice are particularly applicable for studies of HIV infection because human fetal thymus transplants can provide the specific environment in mice for the differentiation of functional human T cells. Using the NSG SGM3 strain, Bryce et al. [74] improved BLT mice to develop large numbers of human FcεRI^+^ mast cells; they successfully induced PCA and passive systemic anaphylaxis via the sensitization of hapten-specific human IgE and the subsequent challenge with hapten in vivo. IgE-independent human mast cell degranulation by C3a, C5a, and Substance P was also observed. The BLT allergy model could become a promising tool for the investigation of mast cell biology and for use in preclinical studies and HSC models. However, BLT mice require human fetal liver and thymus, as well as bone marrow. Therefore, all researchers should consider the technical and ethical concerns involved in developing and using BLT mice. For these reasons, it is difficult to generate BLT mice in countries that limit the use of human-derived tissues. All allergy models using humanized mice introduced in this review are summarized in Table 2.

### 4.5. Humanized Mouse Models of Eosinophilic Airway Inflammation

In this review, we have mainly discussed allergic reactions in the skin and digestive tract of humanized mice. However, immune cells are thought to be crucial for the development of respiratory allergies in humans. Airway allergic inflammatory responses including lymphocytic or eosinophilic inflammation, airway hyperresponsiveness (AHR), and goblet cell hyperplasia are induced by the repetitive inhalation of allergens such as house dust, fungi, and pet hair. [109,110,111,112,113,114]. Many murine models of asthmatic airway inflammation have been established to elucidate the mechanisms of these diseases; however, there are no humanized models, with the sole exception of a PBMC-transferred model. Patient-derived human PBMC-transferred NSG mice have been used for tests of asthmatic airway inflammation [87,115]. In this model, the intratracheal challenge of the allergen causes airway inflammation with AHR [87]. The authors found that HIV-1 envelope glycoprotein 120 (gp120) prevented the condition via Treg cell activation, indicating the therapeutic potential of Treg cells for allergic diseases.

Recently, we examined whether HSC-transferred humanized mouse models can mimic asthmatic airway inflammation. IL-33 is an innate inflammatory cytokine produced by damaged epithelial cells that plays a critical role in the pathogenesis of airway inflammation by inducing the production of type 2 cytokines from IL-33 receptor ST2-expressing cells, such as basophils, mast cells, and type 2 innate lymphoid cells (ILC2) [116,117,118,119]. We intratracheally administered recombinant human IL-33 into NOG IL-3/GM-CSF Tg or NOG IL-3/GM-CSF/IL-5 (triple) Tg mice [120]. These models showed several symptoms of asthmatic diseases, such as inflammation involving human lymphocytes, eosinophils, mast cells, and basophils; the hyperplasia of goblet cells; AHR enhancement; the production of human IL-5, IL-13, and eosinophil-derived neurotoxins; and the secretion of periostin by intratracheal IL-33 administration. In our two HSC-transferred humanized models, human mast and T cells were the major IL-13-producing cells. Several recent studies have demonstrated that ILC2 is a major inducer of eosinophilic airway inflammation when IL-33 is intratracheally administered [116,118]. In a murine model of airway inflammation, Christianson et al. demonstrated that ILC2 was involved in prolonged asthmatic airway inflammation via a positive feedback circuit with lung epithelial cells [116]. Epithelial cell-derived IL-33 affects the production of IL-13 from ILC2. Subsequently, this ILC2 enhances ST2 expression and IL-33 production by epithelial cells. Through this positive feedback mechanism, Th2 cells are redundantly affective instead of ILC2. Thymic stromal lymphopoietin (TSLP) is an important cytokine that enhances allergic reactions and is highly expressed in bronchial epithelial cells in asthma patients [121]. Kabata et al. established a steroid-resistant airway inflammation model that was induced by the simultaneous nasal administration of IL-33 and TSLP. IL-33 stimulation alone elicited IL-5 production from ILC2, and, subsequently, eosinophils infiltrated the airway. This ILC2-dependent reaction was inhibited by corticosteroids to induce apoptotic signals in ILC2. Together with IL-33, TSLP administration significantly suppressed the corticosteroid-induced apoptosis of ILC2; consequently, corticosteroids did not inhibit the airway inflammation via any TSLP mechanisms. The involvement of human ILC2 in this process remains unclear. Further analysis will be necessary to address this issue in future work.

Notably, the human IL-5 transgene greatly enhances the number of circulating human eosinophils in triple Tg mice, even under steady-state conditions. After IL-33 stimulation, the marked infiltration of human eosinophils was induced by murine eotaxin produced from inflamed airway epithelium. Human IL-13 blockade can reduce these inflammatory responses, including eosinophilic inflammation, suggesting that human IL-13 stimulates murine airway epithelium; in turn, epithelium-derived mediators induce these pathologies. Though our new models still have some limitations, we hope that the triple Tg mouse strain will be recognized as a suitable humanized model for preclinical studies of allergic disorders resulting from human immune cell-derived mediators.

## 5. Future Perspectives

In this review, we described about three different approaches for replicating the human immune system for use in allergy models via the transfer of PBMC, HSC, or BLT into immunodeficient mice, each with its own advantages and limitations. As mentioned above, PBMC-engrafted humanized mice are not suitable for a long-term study due the development of xeno-GVHD. Recently, we established two NOG strains, MHC class I and II double knockout mice [122] and human IL-4 Tg mice [123]. Though these strains showed sufficient engraftment of human leukocytes, the development of xeno-GVHD was significantly attenuated after PBMC transplantation. Furthermore, human Th2 cells were the predominant cell type observed in NOG hIL-4 Tg mice following the transfer of naive T cells. The use of these strains may therefore provide a suitable model for long-term study of human allergies using PBMC-transferred humanized mice.

Human myeloid lineage cell-reconstituted second-generation humanized mice transferred with human HSCs are useful models for the study of mast-cell-mediated allergic responses. One limitation of these second-generation humanized mice is that they develop severe anemia, which may be caused by the erythrophagocytosis of mouse red blood cells by human macrophages [124]. Under allogenic conditions, erythrophagocytosis is tightly regulated by the “don’t eat me” signal, mediated by Sirpα on macrophages and the CD47 ligand on red blood cells, and it is an important mechanism of the species barrier [125,126]. This tolerance signal is abolished under xenogenic conditions, particularly in second-generation humanized mice in which human macrophages are significantly differentiated. In the same manner, our NOG IL-3/GM-CSF Tg and NOG IL-3/GM-CSF/IL-5 Tg mice also possessed fewer red blood cells and a lower hemoglobin level and hematocrit ratio beginning four months after HSC transplantation, indicative of persistent erythrophagocytosis. Further research will be necessary to overcome this issue in order to enable the use of second-generation humanized mice in long-term experiments.

The house dust mite (HDM) challenge is the most commonly used procedure to elicit airway inflammation and the production of type 2 cytokines such as IL-33, TSLP, and IL-25 from epithelial cells [127]. In our humanized mice, we intratracheally administered HDM into humanized NOG IL-3/GM-CSF Tg or NOG IL-3/GM-CSF/IL-5 triple Tg mice and analyzed the severity of the airway inflammation. Though many human eosinophils infiltrated the airways of the triple Tg mice, goblet cell hyperplasia and human IL-5 and IL-13 production were not detected [120]. This limitation is explained by a defect in human IL-33 such that HDM does not lead to the production of human IL-33 in these mice and consequently fails to activate human T cells and mast cells. Furthermore, HDM-specific human IgE cannot be produced in these mice due to an intrinsic defect in the class-switching mechanisms in humanized mice [128]. One possible resolution to this problem may be to cross these mice with HLA-expressing Tg NOG mice, which can secrete antigen-specific human antibodies after an antigen challenge when the immune system is reconstituted with HLA-matched human HSC [64].

Various humanized and human monoclonal antibodies (mAbs) have been developed for the treatment of TNF-mediated inflammatory autoimmune diseases and type 2 cytokine-mediated severe allergic disorders. These antibody-based therapies are able to stimulate activity of their target molecules. In the case of anti-TNF biologics, the Fc-regions of anti-membrane TNF mAbs efficiently mediate antibody-dependent cell cytotoxicity (ADCC) by NK cells and complement-dependent cell cytotoxicity [129]. This antibody-mediated cell killing is one of the most important mechanisms of anti-TNF biologics. Anti-type-2 cytokine biologics, such as dupilumab, benralizumab, and mepolizumab, have also been approved for the treatment of severe asthma; however, dupilumab (IL-4Rα antagonist) [130] and mepolizumab (IL-5 antagonist) [131] are not able to induce cell killing. Benralizumab is an anti-IL-5Rα therapeutic antibody that targets human eosinophils in severe and uncontrolled eosinophilic asthma. The antibody is afucosylated to enhance the binding affinity of benralizumab to FcγRIIIa, and it can deplete IL-5Rα-expressing eosinophils and basophils via ADCC [132]. The afucosylation of an antibody results in the enhancement of antibody-dependent cell phagocytosis (ADCP) by macrophages. For instance, MEDI-551, an anti-CD19 afucosylated humanized mAb, exhibits potent in vitro ADCP activity against human precursor-B acute lymphoblastic leukemia [133], although the exact mechanism of this action remains unclear, due in part to the lack of suitable animal models to evaluate the in vivo ADCP activity of benralizumab against eosinophils. Alternatively, instead of NK cells, it is possible to deplete target cells via ADCP in humanized mice [134]. In our triple Tg mice, human NK cells expand poorly, but monocytes differentiate efficiently. Therefore, triple Tg mice may be a useful model for eosinophil-targeted ADCP therapy in in vivo screening systems. Recently, human eosinophils have been reported to exhibit functional heterogeneity between healthy subjects and allergic patients [135]. The characterization of human eosinophils in triple Tg mice may help to elucidate the contribution of eosinophils to these diseases; however, this remains to be attempted. Nasal polyps are an eosinophilic inflammatory disease characterized by accumulated eosinophils, resulting in nasal edema and mucus. Miyata et al. [135] isolated eosinophils from patients with nasal polyps and characterized the features of these inflammatory eosinophils. Nasal polyp eosinophils significantly produced leukotriene D_4_ (LTD_4_) with an increase in the level of gamma glutamine transferase 5 (GGT5), which can metabolize LTC_4_ into LTD_4_. Interestingly, the authors showed that human IL-5- and GM-CSF-stimulated PBMC-derived eosinophils also increased LTD_4_ and GGT5, exhibiting similar phenotypes to nasal polyp eosinophils. Since our triple Tg mice constitutively produce human IL-5 and GM-CSF, we speculate that human eosinophils in triple Tg mice have similar characters and functions to those of nasal polyp eosinophils. Thus, triple Tg mice could be useful as a therapeutic model to target inflammatory eosinophils.

## Figures and Tables

**Table 1:** Specifications of second-generation humanized mice and myeloid lineage differentiation models.

**Strain**		NOG IL-3/GM-CSF Tg	NSG-SGM3 (Tg)	MISTRG (knockin)	NSG-hSCF Tg
**Transgene**		human IL-3. GM-CSF	human IL-3, GM-CSF, SCF	human IL-3, GM-CSF, M-CSF, TPO, SIRPa **	membrane-bound human SCF
**Expression level**		30-100 pg/ml	2,000-4,000 pg/ml *	Not reported	Not reported
**Level of human myeloid cells compared to conventional humanized mice**	**Mono/Macrophage**	Increased	Increased	Increased	No difference
	**Granulocyte**	Eo/Baso increased but Neutro did not	Eo/Baso increased but Neutro did not	Eo/Baso increased but Neutro did not	No difference
	**Mast cell**	Increased	Increased	Not reported	Increased
	**Erythrocyte**	None	None	None	None
	**Platelet**	Slightly increased	Decreased	Not reported	Not reported
**Human disease model**	**Immune diseases**	Allergy, Asthma, Adverse effect of immunotherapy or chemotherapy	Allergy, Macrophage activation syndrome	Not reported	Allergy
	**PDX**	Not reported	AML, MDS, MML	AML, MDS, MM	Not reported
	**Infection**	HIV	Ebora	Listeria, Influenza,	Not reported
**Original publication**		57	55	56	54, 59

* The Jackson Lab. HP (https://www.jax.org/strain/013062) ** Introduced BAC transgene.

**Table 2:** Human allergy humanized mouse models.

Strain	Graft	Induction	Treatment	Reference
NSG	Patient-derived PBMC	Hazelnut-induced allergic gut or airway	-	84
NSG	Patient-derived PBMC	Oxazolone-induced atopic dermatitis	-	85
NSG	Patient-derived PBMC	Allergen-induced allergic gut inflammation	Omalizumab, rGARP, Treg	89
NSG	Patient-derived PBMC	Peanut-induced PCA and PSA	Omalizumab	86
NSG	Patient-derived PBMC	Birch pollen-induced airway inflammation	gp120	87
NOG IL-3/GM-CSF Tg	HSC	hIgE+antigen-induced PCA	C3 antagonist	57
NOG IL-3/GM-CSF Tg	HSC	IL-33-induced airway inflammation	anti-hIL-13 Ab	104
NSG-SGM3	BLT	hIgE+antigen-induced PCA and PSA	-	74
NSG-hSCF Tg	HSC	Peanut-induced PSA	Omalizumab	79

## Abbreviations

AD	Atopic dermatitis
AHR	Airway hyperresponsiveness
BLT	Bone marrow, liver, and thymus
GM-CSF	Granulocyte-macrophage colony stimulating factor
HSC	Hematopoietic stem cell
NOG or NSG	NOD/Shi-*scid* IL2rγ*^null^*
PBMC	Peripheral blood mononuclear cell
PCA	Passive cutaneous anaphylaxis
SCF	Stem cell factor
